# The embryonic leaf identity gene *FUSCA3 *regulates vegetative phase transitions by negatively modulating ethylene-regulated gene expression in Arabidopsis

**DOI:** 10.1186/1741-7007-10-8

**Published:** 2012-02-20

**Authors:** Shelley Lumba, Yuichiro Tsuchiya, Frederic Delmas, Jodi Hezky, Nicholas J Provart, Qing Shi Lu, Peter McCourt, Sonia Gazzarrini

**Affiliations:** 1 Department of Cell and Systems Biology (CSB), University of Toronto, 25 Willcocks Street, Toronto, ON, M5S 3B2, Canada; 2 Department of Biological Sciences, University of Toronto at Scarborough, 1265 Military Trail, Toronto, ON, M1C 1A4, Canada; 3 Centre for the Analysis of Genome Evolution and Function (CAGEF), University of Toronto, 25 Harbord Street, Toronto, ON, M5S 3G5, Canada

**Keywords:** *Arabidopsis*, embryonic development, phase transition, FUSCA3, hormones, ethylene

## Abstract

**Background:**

The embryonic temporal regulator *FUSCA3 *(*FUS3*) plays major roles in the establishment of embryonic leaf identity and the regulation of developmental timing. Loss-of-function mutations of this B3 domain transcription factor result in replacement of cotyledons with leaves and precocious germination, whereas constitutive misexpression causes the conversion of leaves into cotyledon-like organs and delays vegetative and reproductive phase transitions.

**Results:**

Herein we show that activation of FUS3 after germination dampens the expression of genes involved in the biosynthesis and response to the plant hormone ethylene, whereas a loss-of-function *fus3 *mutant shows many phenotypes consistent with increased ethylene signaling. This *FUS3*-dependent regulation of ethylene signaling also impinges on timing functions outside embryogenesis. Loss of *FUS3 *function results in accelerated vegetative phase change, and this is again partially dependent on functional ethylene signaling. This alteration in vegetative phase transition is dependent on both embryonic and vegetative *FUS3 *function, suggesting that this important transcriptional regulator controls both embryonic and vegetative developmental timing.

**Conclusion:**

The results of this study indicate that the embryonic regulator *FUS3 *not only controls the embryonic-to-vegetative phase transition through hormonal (ABA/GA) regulation but also functions postembryonically to delay vegetative phase transitions by negatively modulating ethylene-regulated gene expression.

## Background

Spatial patterning in most multicellular organisms requires genes to both establish regions of cell differentiation and specify cellular fate. In the early *Drosophila *embryo, for example, cells are organized into boundaries by the pair rule and segment polarity genes, then they acquire distinct fates through homeotic gene expression [1]. Homeotic genes are also required to establish boundaries during temporal patterning, whereas heterochronic genes define the timing of the cell fate decisions within those boundaries [2]. One challenge in developmental biology is to identify and understand the overall developmental role of genes involved in the temporal patterning of genetic programs.

Higher plants are well-suited for identifying genes involved in developmental timing because they continually produce easily distinguishable organs throughout the life cycle, whose fates are dependent on the time of emergence [3]. The types of leaves that emerge over time often show distinctive developmental changes that allow them to be classified into juvenile and adult leaves. Later, when a plant enters reproductive development, the vegetative meristematic region switches to an inflorescence meristem that produces flower bracts with floral meristems in their axils [4]. Genetic analysis in *Arabidopsis thaliana *has identified a myriad of genes that converge to control the juvenile to adult leaf transitions and the switch of the vegetative meristem to reproductive development [5].

Unlike flowers and leaves, which form from a shoot apical meristem, the developmental relationship between embryonic leaves (or cotyledons) and adult foliar organs is complicated by cotyledon formation during embryonic patterning. Furthermore, in many plants such as Arabidopsis, cotyledons switch from a storage organ to a more leaflike photosynthetic organ soon after germination. Despite these complexities, single loss-of-function mutations in Arabidopsis have been identified in three genes, *LEAFY COTYLEDON1 *(*LEC1*), *LEAFY COTYLEDON2 *(*LEC2*) and *FUSCA3 *(*FUS3*), whose mutations result in the replacement of cotyledons with organs more similar to vegetative leaves [6-9]. In *lec1 *and *fus3 *mutants, genes that encode markers of late embryogenesis are reduced or missing [9,10]. By contrast, germination markers that normally proceed late embryogenesis are precociously activated. These expression patterns suggest that *LEC1 *and *FUS3 *may establish temporal boundaries. Although little is known about how these genes contribute to temporal patterns, it is known that *FUS3 *regulates and is regulated itself by the synthesis of two terpenoid hormones, abscisic acid (ABA) and gibberellins (GA) [10-12]. The ratio of these two hormones contributes to proper cotyledon patterning by regulating the rates of cell cycling [11].

Although extensive analyses of *LEC1, LEC2 *and *FUS3 *gene action have been carried out with respect to embryogenesis, the effects of these mutations on vegetative leaf development have not been studied extensively [11,13,14]. It has been shown that after germination, the first juvenile leaves of *lec1 *seedlings are shifted toward later leaf identities; however, this shift is not maintained, and successive leaves and flowering time were corrected back to a wild-type pattern [15]. This suggests that embryonic leaf development can have a restricted impact on future vegetative leaf identities. What remains unclear, however, is how cotyledon development impinges on later vegetative development, which is temporally and spatially distinct.

To address such questions, we decided to use a combination of controlled FUS3 activation during vegetative development with whole-genome transcript profiling. Using this approach, we discovered that *FUS3 *downregulates a collection of genes involved in ethylene biosynthesis and signaling. Consistent with this finding, loss-of-function *fus3 *mutants show ectopic ethylene responses at both the developmental and molecular levels. The *fus3 *plants also show precocious vegetative phase change; however, unlike the *lec1 *mutants, this change is not corrected at later adult stages. More importantly, the accelerated vegetative phase transition can be suppressed by inhibiting ethylene action either genetically or pharmacologically. Thus it appears that this previously defined embryonic regulator also has roles in vegetative development. One role of *FUS3 *during early seedling growth is to dampen ethylene action, which in turn contributes to a slowing of subsequent vegetative phase transitions. These results add ethylene to the list of hormones that contribute to temporal patterning in Arabidopsis.

## Results

### Downstream effectors of *FUS3*

**Figure 1:**
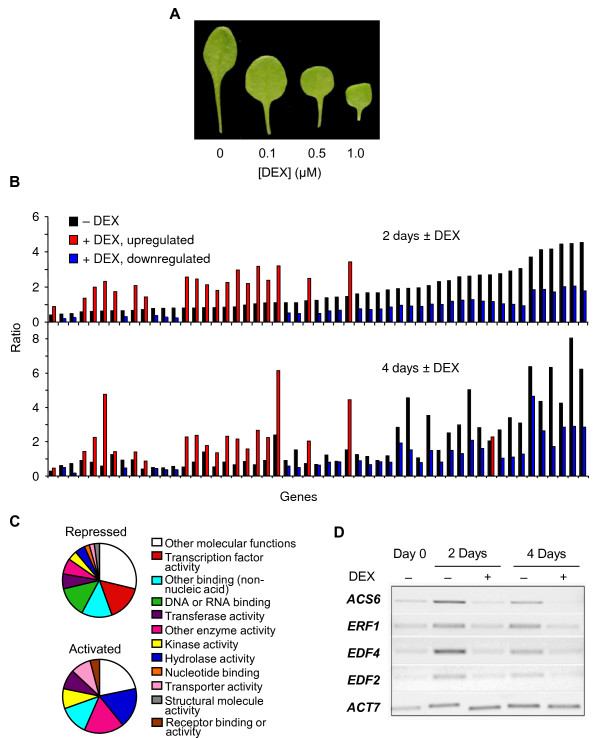
**Microarray analysis of seedlings ectopically expressing *FUS3***. **(A) **Images showing the development of leaf 4 in 5-day-old *fus3 ML1:FUS3-GR *seedlings transferred to different concentrations of dexamethasone (DEX). **(B) **Genes that change at least twofold in expression in *fus3 ML1:FUS3-GR *seedlings treated with 1 μM DEX. The upper graph (2 days ± DEX) shows the ratios of fold changes in gene expression of 5-day-old seedlings grown for 2 days in the absence of DEX (black bars) or in the presence of DEX (red bars represent upregulated genes, and blue bars represent downregulated genes). The same order of genes is represented in the lower graph (4 days ± DEX), where 5-day-old seedlings were grown for 4 days in the absence of DEX (black bars) or in the presence of DEX (red and blue bars). **(C) **The proportions of genes associated with various molecular functions (gene ontology) are represented in the pie chart. **(D) **RT-PCR verification of ethylene-related genes identified by microarray analysis downregulated by FUS3 activation. Expression of four ethylene-related genes and an *ACTIN7 *(*ACT7*) control in the absence (-DEX) or presence (+DEX) of FUS3 activation.

The discovery that *FUS3 *misexpression outside embryogenesis can influence vegetative leaf identity suggests that potential *FUS3*-dependent downstream effectors can be identified through whole-genome microarray analysis [11]. We constructed an inducible misexpression system by transforming the *fus3-3 *(*fus3*) mutant with a *FUS3*-glucocorticoid receptor (GR) translational fusion under the control of the epidermal specific *AtML1 *promoter (*fus3 AtML1:FUS3-GR*) [11]. Transgenic seeds were germinated and grown in minimal medium (Murashige and Skoog, MS) for 5 days, then plantlets were transferred to various concentrations of dexamethasone (DEX) to determine the minimal amount of activation needed to influence leaf identity. All concentrations of DEX tested had an effect on vegetative leaf shape; increasing concentrations of DEX resulted in a leaf with an increased paddle-like shape and a progressively shorter petiole (Figure 1A). At higher than 0.5 μM DEX concentrations, the appearance of trichomes on the upper or adaxial surface of the leaves was completely inhibited (data not shown). On the basis of these conditions, 1.0 μM DEX was chosen as the FUS3-activating condition.

Putative FUS3 targets involved in vegetative phase transitions were identified by examining the transcriptome of seedlings that transiently activate FUS3 using the *AtML1:FUS3-GR *DEX-inducible system [11]. To do this, transgenic seeds from *AtML1:FUS3-GR *were germinated and grown for 5 days in MS. After this time, half of the seedlings were transferred to MS media supplemented with 1.0 μM DEX (+DEX), and the other half were transferred to MS media supplemented with dimethyl sulfoxide (DMSO) (-DEX or control) for 2 or 4 days (Figure 1B). Putative FUS3 targets were chosen on the basis of the following criteria. Genes that increased (red bars) or decreased (blue bars) at least twofold in expression after two days of FUS3 activation (Figure 1B; +DEX) compared to the control (Figure 1B; -DEX) were considered to represent candidate FUS3 targets involved in phase transition. Utilizing this regime and the average of two replicate experiments, 19 genes increased (Additional file 1, Table S1) and 34 genes decreased (Additional file 2, Table S2) in expression (at least twofold) as the direct result of 2-day FUS3 activation (2 days +DEX) compared to the control (2 days -DEX). To independently verify the expression changes of the 2-day DEX induction experiment, a temporal replicate experiment was performed in which FUS3 activation was extended by transferring 5-day-old seedlings onto DEX for 4 days (4 days +DEX). All but 1 of the 53 genes selected by the 2-day experiment exhibited similar expression ratios in the 4-day experiment, demonstrating that activation of FUS3 results in changes in expression levels of a relatively small but reproducible gene set.

Gene ontology (GO) was utilized to categorize the activated and repressed genes by function (Figure 1C). Thirty-eight percent of genes upregulated by FUS3 activation appear to be involved in enzymatic activity, whereas a number of repressed genes are annotated as having roles in hormone synthesis or action. For example, the *CYP707A3 *gene that encodes the cytochrome p450 monooxygenase that catabolizes ABA is repressed more than twofold by FUS3 activation (Additional file 2, Table S2) [16]. This observation, in conjunction with the lack of FUS3-dependent induction of known ABA biosynthetic genes, suggests the increased ABA levels observed previously in FUS3 misexpression lines are due to decreased ABA catabolism [11]. A second class of hormone-related genes that are repressed by FUS3 activation at all time points and in both replicates are either involved in ethylene biosynthesis (*ACS6, ACC synthase 6*) or ethylene response (*ERF1, ETHYLENE RESPONSE FACTOR1*; *ERF104, ETHYLENE RESPONSE FACTOR104*; *ESE3/EREBP, ETHYLENE AND SALT INDUCIBLE3*; *EDF4, ETHYLENE RESPONSE DNA BINDING FACTOR4*). ERFs and EREBPs are transcription factors which contain an AP2 DNA binding domain, whereas EDF (or RAV) transcription factors contain both an AP2 and a B3 DNA-binding domain. Indeed, transcription factors constitute a dominant category (17%) of FUS3-repressed genes (Additional file 2, Table S2).

Potential downstream targets of *FUS3 *would be expected to contain the RY promoter element motif CATGCA, to which the B3 domain of FUS3 binds [17,18]. In fact, the RY sequence was found to be statistically enriched in both the up- and downregulated gene set compared to a randomized sample (*p*-value of 1.77 × 10^-4 ^and 6.06 × 10^-3 ^for 1, 000 bp upregulated and downregulated genes, respectively; Additional files 1 and 2, Tables S1 and S2). Together, these data strongly suggest that our experimental conditions identified a small gene set that is responsive to *FUS3*. Interestingly, this set includes only a few genes that typically mark seed maturation and late embryogenesis, such as seed storage proteins and late embryogenesis abundant proteins (LEA).

### *FUS3 *negatively regulates a subset of genes that are responsive to ethylene

A closer inspection of our microarray data yielded additional ethylene-induced transcription factors (*ERF2, EDF2 *and *EDF1*) whose expression levels were repressed by FUS3 (Additional file 2, Table S2). These genes did not quite meet the stringent criteria of exhibiting a twofold decrease following both 2- and 4-day periods of FUS3 activation. Of the 14 *AP2/EREBP/ERF *and *RAV/EDF *genes that are known to be induced by ethylene exposure, six genes (*ERF1, ERF2, ERF104, EDF2, EDF4 *and *EDF1*) were dampened by FUS3 activation, suggesting that *FUS3 *plays a role in reducing the expression of genes involved in ethylene action [19]. This was verified by RT-PCR performed on *ACS6, ERF1, EDF4 *and *EDF2 *at 2 and 4 days of DEX induction (Figure 1D).

**Figure 2:**
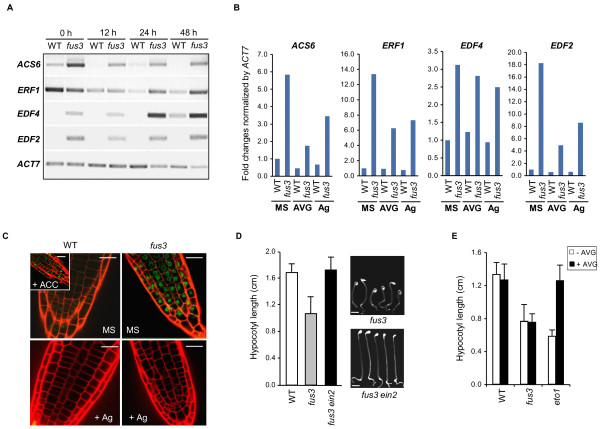
**Increased expression of ethylene signaling and biosynthetic genes in *fus3 *mutant, and ethylene-related *fus3 *phenotypes**. **(A) **RT-PCR of ethylene-related genes in wild-type (WT) and *fus3 *seeds germinated for 12, 24 and 48 hours in minimal medium (MS). *ACTIN7 *(*ACT7*) served as a control. **(B) **RT-PCR of ethylene-related genes in wild-type and *fus3 *seeds germinated for 48 hours in MS media or MS media containing 10 μM aminoethoxyvinylglycine (AVG) or 100 μM AgNO_3 _(Ag). Fold change in gene expression was normalized to *ACTIN7*, which served as a control. Similar trends were seen in two independent experiments. **(C) **GFP-EIN3 fluorescence in wild-type and *fus3 *roots incubated for 48 hours in minimal medium with (+) or without (-) 100 μM AgN0_3 _(Ag). The inset shows the GFP-EIN3 fluorescence in wild-type roots exposed to the ethylene precursor 1-aminocyclopropane-1-carboxylic acid (ACC). Scale bar = 15.2 μm. **(D) **Images (right panels) and quantification (left panel) of hypocotyls length of 5-day-old *fus3 *and *fus3 ein2-1 *seedlings grown in the dark in the air. Scale bar = 0.2 cm. Averages from triplicate experiments ± SD are shown. *fus3, n *= 24; *fus3 ein2-1, n *= 21. **(E) **Quantification of hypocotyls length of 5-day-old wild-type, *fus3 *and *eto1-1 *seedlings grown in the dark in MS in the absence (-) or in the presence (+) of the ethylene synthesis inhibitor AVG. The *eto1-1 *seed, which overaccumulates ethylene gas, was used as a positive control to demonstrate rescue by AVG addition. Averages from triplicate experiments ± SD are shown. Wild type, *n *= 25; *fus3, n *= 25; *eto1-1, n *= 24.

FUS3-mediated repression of genes involved in ethylene synthesis and action predict that *fus3 *loss-of-function mutants would exhibit an inverse expression pattern of these genes. Indeed, expression of *ACS6, EDF2 *and *EDF4 *genes were consistently higher in germinating *fus3 *versus wild type at all time points surveyed after imbibition, whereas *ERF1 *expression increased after 24 hours (Figure 2A). These results again support a negative role for *FUS3 *in regulating the expression of these ethylene responsive genes during germination. The presence of the RY element in the promoters of this gene subset may mean FUS3 directly downregulates their expression. Alternatively, *FUS3 *may act through ethylene synthesis or signaling to modulate their expression. To test these possibilities, we repeated the RT-PCR analysis of *ACS6, ERF1, EDF4 *and *EDF2 *on 2-day old wild-type and *fus3 *seedlings germinated in the presence of either an ethylene biosynthesis inhibitor, aminoethoxyvinylglycine (AVG), or an ethylene signaling inhibitor, silver ions (AgNO_3_). In both cases, the inhibitors partially suppressed the increased levels of the gene transcripts in *fus3 *seedlings compared to untreated controls (Figure 2B). Furthermore, a key transcriptional regulator of ethylene signaling, EIN3 [20,21], showed increased protein stability in the emerging root cells of *fus3 *compared to wild type, and this stability was eliminated by the addition of silver ions (Figure 2C). Although these results do not exclude a direct role for *FUS3 *on ethylene-responsive gene transcription, it does suggest that at least part of the aberrant gene expression observed in *fus3 *mutants does require functional ethylene synthesis or signaling.

To further probe the connection of *FUS3 *with ethylene action during germination, we studied the growth of dark-grown *fus3 *loss-of-function seedlings. When wild-type seedlings are germinated in the dark in the presence of ethylene, they show an exaggerated apical hook, a shortening of the hypocotyl and reduced root growth [22]. Termed the "triple response, " this developmental assay has been extremely useful in genetically dissecting the role of various genes involved in ethylene synthesis or signaling in Arabidopsis [23]. Dark-germinated *fus3 *plantlets grown in the absence of ethylene exhibited shorter hypocotyl growth and hooked cotyledon development (Figure 2D), which is consistent with their ectopic ethylene gene expression. Moreover, these phenotypes were alleviated by the introduction of a mutation (*ein2-1*) that reduces ethylene signaling into the *fus3 *genetic background (Figure 2D). To further differentiate ethylene synthesis and response, we repeated the experiment in the presence of the ethylene biosynthesis inhibitor AVG. In contrast to the *ein2 *mutation, AVG did not restore the hypocotyl length of *fus3*, which suggests that the ectopic ethylene responses observed in dark-grown *fus3 *seedlings were not due to increased ethylene synthesis (Figure 2E).

### *FUS3 *functions to repress ethylene action during germination

The heightened ethylene responses observed in *fus3 *after germination suggest that loss of this gene function might have other, uncharacterized effects on vegetative development. Because *FUS3 *is a regulator of embryonic leaf identity, we decided to examine the vegetative leaf identity and phase transitions of the *fus3 *loss-of-function mutant more closely. From the emergence of the first leaf after germination through to flowering, each wild-type leaf adopts a unique identity based on size and shape, and this graded growth variation is often referred to as the "leaf heteroblastic series" [23]. Although rosette leaves always produce trichomes on the adaxial side of the wild-type leaf, trichomes on the abaxial side begin to appear on leaves only at later nodes (leaf 5 in wild type). Leaves that produce only adaxial trichomes are considered juvenile, whereas later leaves that show trichomes on both sides are regarded as adult [24]. Also, the ratio of leaf blade-to-petiole lengths is generally lower in early juvenile leaves and becomes higher as the plant transitions to adult leaves and flowering [25].

**Figure 3:**
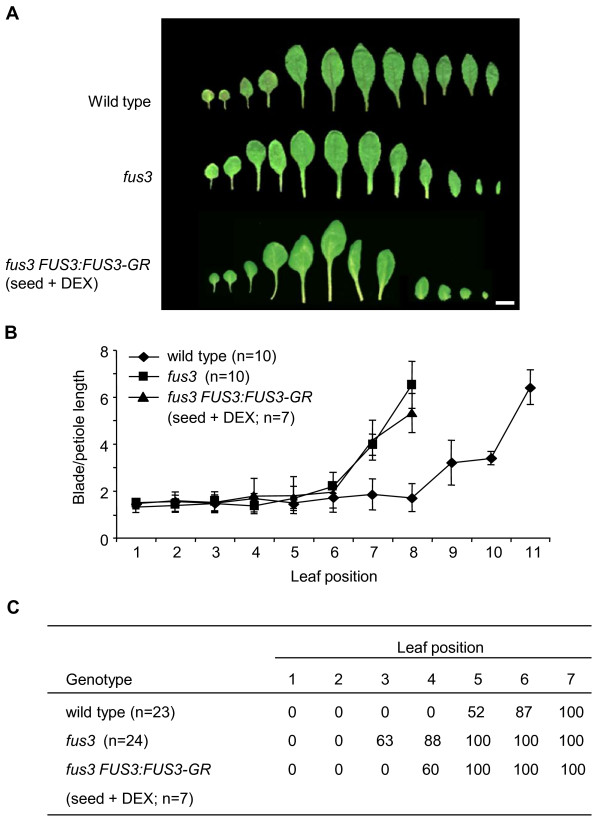
**Contributions of embryonic *FUS3 *to leaf identity and phase transitions**. **(A) **Rosette leaf morphology of wild-type (top row), *fus3 *(middle row) and *fus3 *plants transformed with the *FUS3:FUS3-GR *construct (bottom row). *fus3 FUS3:FUS3-GR *parent plants were sprayed with 30 μM dexamethasone (DEX) during seed production. Leaves from ten plants were dissected, and representative profiles are shown (scale bar = 1 cm). **(B) **Ratios of blade-to-petiole lengths of individual rosette leaves in wild-type and *fus3 *and embryonically rescued *fus3 FUS3:FUS3-GR *plants. **(C) **Percentage of wild-type, *fus3 *and embryonically rescued *fus3 FUS3:FUS3-GR *(seed + DEX) rosettes that developed an abaxial trichome at each leaf position.

Comparisons of leaf profiles between the *fus3 *mutant and wild-type plants showed that the first two leaves from the mutant plant were more similar in shape and size to the third and fourth leaves of the wild type, and that this shift continued throughout the heteroblastic series (Figure 3A). This precocious shift was reflected in the blade-to-petiole length ratio of each individual leaf (Figure 3B) and in the leaf trichome distribution (Figure 3C). Abaxial trichomes frequently (63%) appeared on leaf 3 in *fus3 *mutant plants, approximately two leaves earlier than the wild type.

### *FUS3 *has functions outside embryogenesis

**Figure 4:**
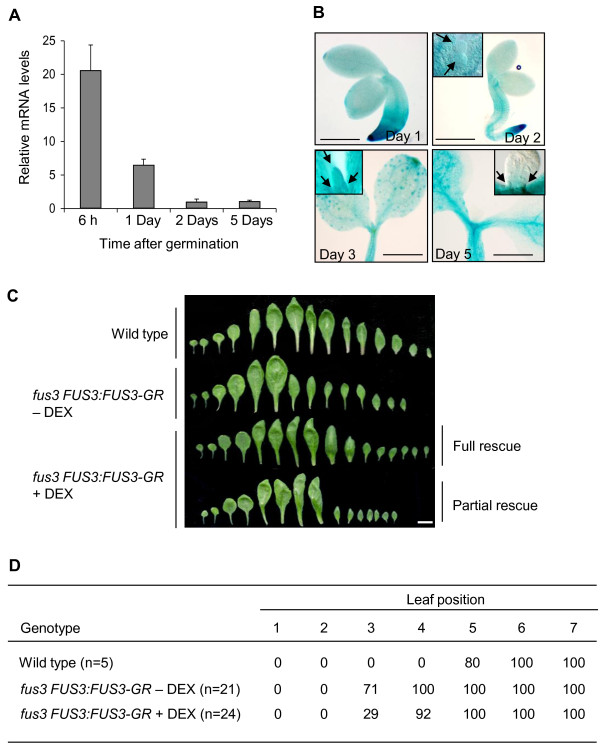
***FUS3 *is expressed and functions postembryonically**. **(A) **Relative expression of the *FUS3 *gene at various time points after germination. Quantitative RT-PCR was performed on germinating wild-type seeds imbibed for 6 hours, 1 day, 2 days and 5 days in minimal medium (MS). Transcript levels were normalized using *ACTIN7 *as an internal control. Results from triplicate samples are shown with error bars (SD). Experiments were repeated twice with similar results. Data from one of these replicates are shown. **(B) **Histochemical staining of *FUS3:GUS *seedlings at 1, 2, 3 and 5 days after germination in MS. Arrows indicate emerging leaf primordia showing β-glucuronidase (GUS) staining. Scale bars = 0.2 mm (day 1) and 0.4 mm (days 2, 3 and 5). **(C) **Wild-type and *fus3 FUS3:FUS3-GR *seeds germinated in MS without dexamethasone (DEX) (-DEX) or on 10 μM DEX (+DEX) for 2 days, then transferred to soil until bolting. Of the plants analyzed, 8.3% showed a full rescue of the rosette leaf morphology, and an example is shown (full rescue). A partial rescue was obtained for 62.5% of the plants, and an example is shown (partial rescue). The morphologies of the first six to eight *fus3 FUS3:FUS3-GR *leaves were rescued, but those of subsequent leaves were variable. Twenty-one to twenty-four plants were analyzed, constituting a representative profile. Scale bar = 1.0 cm. **(D) **Percentage of abaxial trichomes in wild-type rosettes and in *fus3 FUS3:FUS3-GR *rosettes of plants grown with (+) or without (-) DEX.

There are two explanations for the vegetative phenotypes of the *fus3 *mutant. Possibly the altered cotyledon development during embryogenesis in the mutant advances the progression of vegetative leaf identities after germination. Alternatively, *FUS3 *may function in the developing vegetative meristem. We tested these possibilities directly by measuring *FUS3 *transcript levels using quantitative RT-PCR (qRT-PCR). In wild-type seeds, *FUS3 *transcript is detected 6 hours postimbibition and declines over time, as previously shown [26]; it is still detectable 5 days after germination (Figure 4A). To further clarify the source tissue of postembryonic *FUS3 *expression, a transgenic line containing a sensitive *FUS3:GUS *transcriptional fusion was germinated and sampled over time for β-glucuronidase (GUS)-dependent blue histochemical staining [27]. The *FUS3:GUS *line used in these experiments was previously confirmed to reliably report embryonic *FUS3 *expression patterns based on *FUS3 in situ *hybridization [27]. Whole-mount preparations of seedlings showed blue staining in emerging leaf primordia from 2- to 5-day-old seedlings (Figure 4B). A similar expression pattern was found using a *FUS3:GFP *reporter previously described [26] (data not shown).

To functionally determine the effect of *FUS3 *from embryonic and vegetative tissues on the precocious vegetative phase transition, we constructed transgenic plants in which FUS3 could be activated either in the embryo or in vegetative tissue. *fus3 *was transformed with a *FUS3*-*GR *translational fusion construct that was under the control of the native *FUS3 *promoter (*fus3 FUS3:FUS3-GR*). Seeds produced from these lines in the absence of DEX application were phenotypically indistinguishable from *fus3 *loss-of-function mutants, with approximately 95% of the seed being desiccation-intolerant after 6 weeks of storage (data not shown). Of the small number of seeds that did survive desiccation, all produced ectopic trichomes on their cotyledons, as expected. Thus, in the absence of DEX application, the *FUS3-GR *fusion protein cannot rescue any of the known embryonic *fus3 *phenotypes. By contrast, in two independent experiments, approximately 50% of the seed produced by transgenic plants sprayed with 30 μM DEX during flowering showed desiccation tolerance after 6 weeks of storage. Of these, approximately 85% lacked ectopic trichomes on the cotyledons. The large reduction of *fus3 *seed phenotypes indicates that DEX application during flowering is sufficient to rescue mutant embryos.

From the transgenic seeds that were rescued, seven independent plants were randomly selected and grown to maturity in the absence of DEX, and leaf profiles were performed. Leaf profiles of all these lines still displayed accelerated heteroblastic development similar to that observed in *fus3 *leaf profiles (Figure 3A). Furthermore, the lines showed the same advanced vegetative phase transition as *fus3 *with respect to both individual leaf blade-to-petiole ratios (Figure 3B). The abaxial trichomes, however, did show a partial reversion to a wild-type profile (Figure 3C). Together, these results suggest that specifically removing embryonic *FUS3 *has a limited influence on the development of the first two juvenile leaves of Arabidopsis, but not on later leaves.

The inability of embryonic *FUS3 *to fully rescue the precocious vegetative phase transitions of *fus3 *suggests that this gene may function outside seed development. We tested this possibility by using the *fus3 FUS3:FUS3-GR *transgenic lines. Unlike in the previous experiment, however, we allowed transgenic lines to produce seed in the absence of DEX application and then germinated the seed in the presence of 10 μM DEX for 2 days. This short DEX application did not influence the heteroblastic phase change in wild-type plants as measured by either the blade-to-petiole transition or the appearance of abaxial trichomes in comparison to untreated plants (Additional file 3, Figure S1). By contrast, exposure of germinating *fus3 FUS3:FUS3-GR *transgenic seeds to DEX for 2 days resulted in a partial (62.5%) or full (8.3%) shift of leaf heteroblasty back toward a wild-type profile (Figure 4C). This rescue was partially reflected in trichome appearance (Figure 4D) and in full-leaf measurements; in wild type, the largest leaf was leaf 7, reaching a size of 3.9 ± 0.2 cm (*n *= 24). Similarly, the largest leaf in *fus3 FUS3:FUS3-GR *plants treated with DEX was leaf 7, which reached a size of 3.6 ± 0.3 cm (*n *= 23). In contrast, leaf 6 was the largest leaf in *fus3 FUS3:FUS3-GR *untreated plants (3.0 ± 0.3 cm; *n *= 23). In conclusion, a short pulse of FUS3 during the first 2 days of postembryonic growth can influence leaf identity in juvenile leaves.

### *FUS3*-dependent vegetative phase variation requires functional ethylene signaling

**Figure 5:**
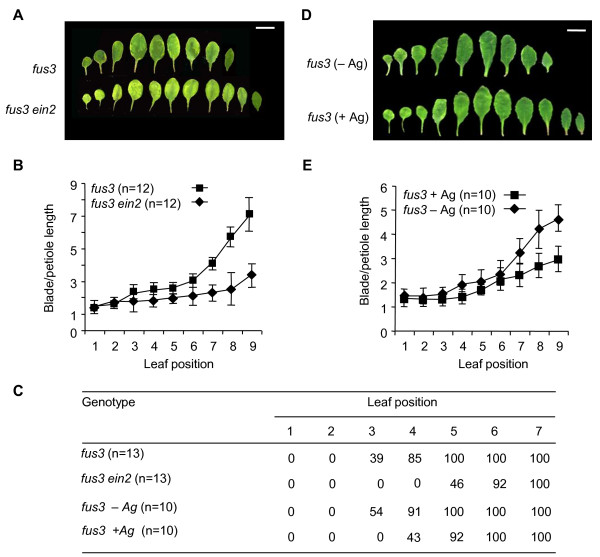
**Influence of ethylene signaling on *fus3 *vegetative phase transition**. **(A) **Mature rosette leaf morphologies of *fus3 *and *fus3 ein2-1 *plants. Leaves from 12 plants were dissected, and a representative profile is shown. Scale bar = 1.0 cm. **(B) **Ratios of blade-to-petiole lengths of mature individual rosette leaves from *fus3 *and *fus3 ein2-1 *plants. **(C) **Percentage of rosettes displaying trichomes on the abaxial surface at each leaf position. **(D) **Rosette leaf morphology of *fus3 *germinated for 2 days in minimal medium (MS) in the presence (+) or absence (-) of 100 μM AgNO_3 _(Ag) and then transferred to soil. Leaves from ten plants were dissected, and a representative profile is shown. Scale bar = 1.0 cm. **(E) **Ratios of blade-to-petiole lengths of individual *fus3 *rosette leaves of plants germinated in MS with (+) or without (-) 100 μM AgNO_3 _(Ag).

Loss-of-function *fus3 *mutants show advanced vegetative phase transition, increased expression of ethylene-regulated genes and phenotypes that are characteristic of increased ethylene signaling. To test if these phenotypes are related, a mutation conferring ethylene insensitivity was introduced into the *fus3 *background. The introduction of the *ein2 *mutation did suppress defects in vegetative phase change compared to the *fus3 *single mutant as measured by leaf profiles, blade-to-petiole ratios and abaxial trichome appearance (Figures 5A to 5C). These results suggest that increased ethylene signaling does contribute to the advanced vegetative phase transition phenotype observed in *fus3*.

To further refine the ethylene contribution, we germinated *fus3 *seeds on 100 μM AgNO_3 _for 2 days and transferred the resulting seedlings to soil. Again, the *fus3 *phase transition phenotypes in the presence of AgNO_3 _were similar to those seen in the *fus3 ein2 *double-mutant (Figures 5C to 5E). Because in this experiment *fus3 *embryonic development occurred in the absence of the ethylene signaling inhibitor AgNO_3_, any alterations in *fus3 *vegetative phase transition were due to the transient inhibition of ethylene signaling during germination. In conclusion, the inhibition of ethylene signaling during early germination is able to partially suppress the premature vegetative phase transitions observed in the *fus3 *mutant.

## Discussion

### The *FUS3 *hormonal framework

Four lines of evidence suggest that *FUS3 *negatively regulates ethylene action. First, gain-of-function activation of FUS3 dampens the expression of a collection of ethylene-responsive genes, whereas a loss-of-function *fus3 *allele shows the opposite effect. Second, the ectopic expression of these ethylene-responsive genes in light-grown seedlings is dampened by the addition of ethylene synthesis or signaling inhibitors. Third, loss-of-function *fus3 *seedlings show common ethylene-response phenotypes in the dark which are dependent on ethylene signaling and also have increased stability of the key positive regulator of ethylene signaling, *EIN3*. Finally, the precocious vegetative phase variation observed in *fus3 *mutants is suppressed if ethylene signaling is inhibited genetically or pharmacologically.

Inhibition of ethylene biosynthesis does not rescue *fus3 *phenotypes in the dark, suggesting that alteration of ethylene signaling in *fus3 *is not merely a consequence of increased ethylene levels and requires functional ethylene signaling. However, genetic manipulation of *FUS3 *did influence the expression of *ACS6*, encoding for an enzyme involved in ethylene biosynthesis. The altered expression of this gene might reflect a feedback response to changes in ethylene signaling. On this note, only a subset of ethylene signaling genes was regulated by *FUS3*, which suggests that the relationship between *FUS3 *and ethylene is complex. Many of the ethylene-responsive genes regulated by *FUS3 *contain RY elements in their promoter, which suggests that this transcription factor may bind these promoters directly. A direct regulation of gene transcription may explain why the addition of ethylene inhibitors did not fully alleviate the ectopic expression of the ethylene-responsive genes. Nevertheless, the negative effect of *FUS3 *on the regulation of ethylene signaling adds this hormone to ABA and GA as those that are dependent on FUS3 during germination [11].

Coordination of the synthesis and signaling of various hormones is important in regulating overall plant growth and development, and many examples of ethylene, ABA and GA interactions have been reported [28-33]. In one of the best-studied cases, initial submergence of deepwater rice plants resulted in the accumulation of ethylene, which in turn enhanced GA sensitivity of the internodes to promote rapid growth [29]. The addition of ABA had the opposite effect by decreasing the sensitivity of internode tissues to GA [32]. With respect to leaf identity, interactions between ethylene and ABA have also been implicated in semiaquatic plants that exhibit heterophylly [34]. ABA induces terrestrial leaf development and ethylene stimulates the formation of submerged leaves in part by reducing ABA levels [30]. In Arabidopsis, where genetic analysis can be applied, loss of ethylene response does increase ABA levels in leaves, and, conversely, Arabidopsis mutants deficient in ABA synthesis show increased ethylene production [35-38]. The antagonistic and seemingly common relationships between ABA and GA and between ABA and ethylene imply that *FUS3 *acts as a control point of these multiple hormone pathways.

Suppressing ethylene signaling in *fus3 *mutants partially rescued the defects in juvenile leaf identities and the accelerated vegetative phase variation of this mutant, but did not rescue the embryonic leaf identity phenotypes. Perhaps this is not surprising, because there are major cellular differences beyond size and shape between embryonic cotyledons and juvenile and adult leaves. For example, genetic programs involved in storage reserves and desiccation appear to be embryo-specific. Related to this is that controlled FUS3 activation outside seed development was not sufficient to upregulate most of the standard seed-specific marker genes such as storage proteins, suggesting that other regulators are also required. The absence of seed-specific marker induction by the controlled activation of FUS3 in seedlings is similar to results obtained by Kagaya *et al*. [39], who also utilized a transgene-based activation system. Under their study conditions, vegetatively activated FUS3 could induce ectopic seed storage gene expression only with the addition of ABA. These observations are in contrast to constitutive FUS3 misexpression experiments, which result in storage reserve accumulation in vegetative tissues. Genetically reducing ABA levels in plants that constitutively express *FUS3 *also attenuates the ability of FUS3 to activate embryonic programs ectopically, consistent with an ABA requirement for full FUS3 function [11]. It appears that there is a limited time during germination during which tissues are more sensitized to FUS3 action. The presence of the transcription factor *ABI5 *has been posited to define a 60-hour developmental window during germination during which ABA can still arrest the growth of the emerging embryo and induce late-embryogenesis gene expression [40]. Whether this 60-hour checkpoint also defines a window in which FUS3 can reactivate late embryogenesis programming needs to be determined, but our expression studies place postembryonic *FUS3 *within this developmental window.

### *FUS3 *and vegetative phase change

Transitions from one leaf identity to the next depend on the coordination of at least two independent processes: the timing of leaf initiation and a program that determines the duration of a developmental phase [3]. In the latter case, the genes that define the juvenile boundary may repress the expression of adult leaf-promoting genes above a certain threshold. One prediction of this model is that these juvenile boundary regulators will decrease in activity over time to allow adult phase change to occur [41]. *FUS3 *transcripts are detected after germination, and although they decrease, they can still be detected 5 days postgermination at a time when at least four to five leaf primordia have formed [42]. Because leaf 5 is considered to be the first to show adult characteristics, the decreased temporal domain of *FUS3 *expression correlates well with decreasing juvenile leaf identity. A simple model would suggest that the presence of FUS3 in leaf primordia contributes to juvenile leaf identity and also that as FUS3 activity drops, the transition to adult leaf phases can occur.

Although we have shown a vegetative role for *FUS3*, embryonic expression of *FUS3 *also affects the leaf identity of the first two juvenile leaves. This is similar to studies involving *LEC1*, which suggests that the identity of the embryonic foliar organs influences the identity of subsequent vegetative organs despite their different developmental origins [15]. Unlike *fus3 *mutants, however, aberrant vegetative phase transitions in *lec1 *plants are corrected in later leaves [15]. The lack of a detectable *LEC1 *signal after germination, as shown in public microarrays [43], may explain the difference between these mutants.

**Figure 6:**
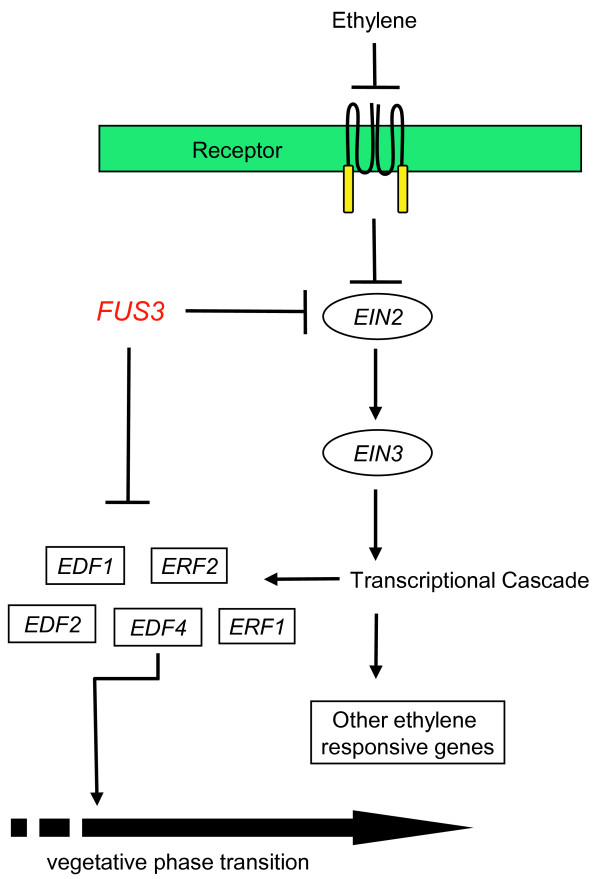
**Working model of phase change regulation by *FUS3***. During late embryogenesis and early germination, *FUS3 *negatively influences a number of factors, including ethylene signaling. As a consequence, the EIN3 protein, a key positive regulator of ethylene signaling, is reduced, thereby causing a decrease in the expression of downstream transcription factors such as *ERF*s and *EDF*s. A reduction of these ethylene-dependent transcription factors prevents the premature transition from the juvenile to the adult phase of development. In the loss-of-function *fus3 *mutant, ethylene signaling increases, which in turn accelerates vegetative phase transitions. The lack of full restoration of altered gene expression by ethylene inhibitors and the observation that only a subset of ethylene responsive genes are affected by *FUS3 *also suggest that *FUS3 *may have ethylene-independent effects. Consistent with this hypothesis, many ethylene-responsive genes contain FUS3-binding RY sequences in their promoter elements. It is therefore possible that *FUS3 *also influences these genes and some aspects of phase transitions through ethylene-independent mechanisms.

In contrast to animals, plant hormones can function in many different tissues and at various times during development, resulting in various developmental outcomes. One of the mechanisms by which *FUS3 *prolongs the juvenile phase appears to involve the downregulation of ethylene action, which suggests that this hormone is important in promoting adult-phase transitions (Figure 6). Morphometric analysis of leaf growth of *etr1 *mutants have shown that this ethylene-insensitive plant produce shorter, broader leaves, which are more akin to a juvenile leaf identity [37]. Consistent with this, the vegetative-to-floral transition in a collection of ethylene-insensitive mutants is delayed compared to the wild type [44]. In agreement with this, *ML1:FUS3-GFP *plants that constitutively express *FUS3 *postembryonically also show delayed flowering [11,26]. Using ethylene to quicken vegetative phase transitions is consistent with the role of this hormone in promoting germination [36,38]. Perhaps the short vegetative pulse of *FUS3 *serves to dampen the action of this hormone after it has stimulated germination so that the leaf identities do not advance too quickly.

## Conclusions

Herein we provide evidence that the embryonic regulator *FUS3 *also plays an important role postembryonically by negatively regulating vegetative phase transitions through repression of ethylene action. This study also implicates a role for ethylene in temporal patterning. Together with previous findings showing that FUS3 controls the embryonic-to-vegetative phase transition by modulating the ABA/GA ratio, this highlights a pivotal role of FUS3 in controlling the timing of expression of embryonic and vegetative programs through hormonal regulation. The pivotal role of FUS3 in regulating hormone levels and responses introduces another dimension to understanding the transitions of leaf identity that occur throughout the plant's life cycle. ABA and ethylene levels are also very dependent on both abiotic and biotic external signals, such as water availability, temperature and pathogen attack. Hence, the phase transitions observed will be dependent not only on developmental regulators such as *FUS3 *but also on environmental changes that influence hormone concentrations. In the future, it will be interesting to see if some of phenotypic plasticity observed in leaf shape can be linked not only to the relative timing of developmental regulators like *FUS3 *but also to environmental conditions, both of which impinge on ethylene, ABA and GA synthesis and/or signaling.

## Methods

### Plant material, growth conditions and leaf profiles

*fus3-3 *(*fus3*) [7] and all other strains used in this study were derived from a Columbia (Col) genetic background. For all experiments, plants were grown at 20°C under constant light. Seeds from all genotypes were generated from parent plants grown under identical conditions and stored under the same conditions for the same length of time. All transformations were performed as previously described [45]. DEX (Sigma-Aldrich, St Louis, MO, USA) was dissolved in DMSO. We performed leaf profiles according to Telfer *et al*. [24], for which 10 to 15 seedlings were germinated and grown in individual 4-inch round pots for approximately 3.5 weeks. To determine if DEX had any off-target effects on leaf heteroblasty, we performed blade-to-petiole measurements and trichome appearance assays on 10 wild-type plants and found that the chemical did not influence either of these markers of phase transition (Additional file 3, Figure S1). For the *ein2 fus3 *double-mutant construction, *ein2-1 *homozygotes were identified by screening for an altered triple-response phenotype in a segregating F2 population. Positive *ein2-1 *plants were grown and assayed for *fus3 *allele using a cleaved amplified polymorphic sequence marker that marks the mutant polymorphism [11]. For the pharmacological studies, 100 μM of the inhibitor AgNO_3 _or 10 μM of the inhibitor AVG were added to MS plates.

### Cloning and generation of transgenic plants

The *EIN3 *cDNA was amplified and carboxy-terminally fused to *GFP *under the control of the 35S promoter in the pEGAD vector using the primers EIN3-*Bam*HI forward 5'-ATA GGA TCC ATG ATG TTT AAT GAG ATG GGA AAT G-3'; EIN3-*Bam*HI reverse 5'-ATA GGA TCC GAA CCA TAT GGA TAC ATC TTG C-3'; and the *Bam*HI enzyme [46] to generate *35S:GFP-EIN3*. The *GFP-EIN3 *translational fusion reporter fully rescues *ein3 *loss-of-function ethylene-dependent phenotypes (data not shown).

### RT-PCR analysis

Thirty PCR cycles were performed with 250 ng of total RNA to amplify ethylene-related genes identified through microarrays using the following primers: *ERF1 *forward 5'-GTA TCC TCA ACG ACG CCT TTC AC-3'; *ERF1 *reverse 5'-CTT CAC CGT CAA TCC CTT ATC C-3'; *EDF4/RAV1 *forward 5'-GTA CAG GTT CCA TCT GTG AAA CC-3'; *EDF4/RAV1 *reverse 5'-CTC GTC TTC GTC CAT CTT CAC GTC-3'; *EDF2/RAV2 *forward 5'-GCA TAG ACG AGA TAA GTT CCT CCA C-3'; *EDF2/RAV2 *reverse 5'-GTT CTT GAA GTT GAC GAC GGC GTC G-3'; *ACS6 *forward 5'-ACA TCC AGA AGC TTC GAT TTG TAC-3'; and *ACS6 *reverse 5'-CTC GTC TAC AAA CTC TTC ATC GGA-3'. These primers were also used to check for expression of genes involved in ethylene synthesis and signaling in dry wild-type (Col) and *fus3 *immature seeds. *ACTIN7 *was amplified using the following primers: *ACT7 *forward 5'-GGT GAG GAT ATT CAG CCA CTT GTC TG-3' and *ACT7 *reverse 5'-TGT GAG ATC CCG ACC CGC AAG ATC-3' and was used as the control. Seeds were imbibed as described for 12, 24 and 48 hours.

### Quantitative RT-PCR

RNA extraction and qRT-PCR were performed as previously described [26]. Sequences of gene-specific primers were designed to amplify 190-bp products. These included the following: ACT7_190 _forward 5'-TCA CAG AGG CAC CTC TTA ACC-3'; ACT7_190 _reverse, 5'-CCC TCG TAG ATT GGC ACA G-3'; FUS3_190 _forward, 5'-TGT GAA TGC TCA TGG TCT GC-3'; and FUS3_190 _reverse, 5'-GGA GGA GAA GAT CGT TAA CCA C-3'.

### Histochemistry and microscopy

Detection of GUS activity and sectioning was performed as described by Donnelly *et al*. [47]. Leaf tissues were cleared in 70% ethanol and 8:2:1 chloral hydrate:glycerol:water, then mounted in the same solution on microscope slides.

### Microarray and bioinformatic analysis

Wild-type Columbia seeds containing the *ML1:FUS3:GR *construct [11] were imbibed for 5 days at 4°C on filter paper placed on MS plates. To minimize the effects of biological variation, we followed a pooling strategy according to that described by Zhu *et al*. [48] in which approximately 100 individual plants per replicate experiment were harvested and pooled before RNA extraction and hybridization. The seedlings were then transferred to 20°C for 5 days. Shoot tissues from seedlings were collected for RNA isolation (day 5). The remaining seedlings on the filter paper were transferred to either MS plates consisting of 1 μM DEX or the same concentration of DMSO. Two and four days after the transfer, shoot tissue was collected from the seedlings. The above protocol was repeated to obtain a second biological replica.

The standard Affymetrix labeling and hybridization protocols were used on an ATH1 Affymetrix GeneChip microarray encompassing 22, 814 probe sets (Affymetrix Inc, Santa Clara, CA, USA). Data were globally normalized using the MAS5.0 global normalization algorithm with a target value of 500. The resulting data were filtered to eliminate MAS5.0 "marginal" and "absent" calls (Present requires both sets of replicates for treatment and control samples). In addition, the *r *values for the correlation of the expression values between the two replicate set of experiments was greater than 0.95. The complete data set has been submitted to the EMBL-EBI ArrayExpress database under the accession number E-MEXP-3465 in compliance with MIAME standards.

The average expression value for each gene was calculated for two replicate samples at each time point. The Cluster and TreeView software programs were used for cluster analysis [49]. The data set was filtered to identify genes that exhibited more than a twofold increase or decrease in expression level [50]. Cluster analysis was performed using average linkage hierarchical clustering with the centered Pearson correlation coefficient as the similarity metric. RY motif (CATGCA) and *p*-values were obtained using the "Motif Analysis" tool on TAIR http://www.arabidopsis.org/tools/bulk/motiffinder/index.jsp.

### Temporal activation of FUS3

The *FUS3:FUS3-GR *construct was made by replacing the *ML1 *promoter in the *ML1:FUS3-GR *construct with the *FUS3 *promoter from the *FUS3:FUS3-GFP *construct previously described [11]. For FUS3:FUS3-GR activation during seed development, DEX was diluted to 30 μM in dH_2_O containing 0.05% Triton X-100 and sprayed on flowering plants twice weekly until the plants had senesced.

### Confocal microscopy

To study EIN3 stability in a *fus3 *background, we crossed *fus3 *with an *ein3 35S:GFP:EIN3 *transgenic line. The stability of the EIN3 protein in the *fus3 *background was compared to non-*fus3 *mutant plants from the same F1 plant. At least 20 seedlings were observed for each genotype. A Nikon inverted fluorescence microscope equipped with a Nikon water immersion objective (Nikon Instruments, Inc, Melville, NY, USA) and a Bio-Rad Radiance 2000 confocal head (Bio-Rad Laboratories, Hercules, CA, USA) was used to detect GFP fluorescence. The same confocal settings were used in all experiments.

## Abbreviations

bp: base pair; GFP: green fluorescent protein; RT-PCR: reverse transcriptase polymerase chain reaction.

## Competing interests

The authors declare that they have no competing interests.

## Authors' contributions

PM and SG designed the experiments and wrote the manuscript. SL and SG performed the biological part of the microarray study and the data analysis of the microarray experiment (Figure 1 and supplementary tables). SL and YT performed RT-PCR. SL performed the experiments shown in Figures 3 and 5. QSL and SG performed the experiments shown in Figure 4. FD and JH constructed and selected *35S:GFP-EIN3 *transgenics. YT constructed and selected FUS3-GR constructs. NJP performed cluster analysis. All authors read and approved the final manuscript.

## Supplementary Material

Additional file 1 **Table S1 Genes upregulated by ectopic FUS3 activation**. Values for fold changes in expression after 2 and 4 days (d) of FUS3 activation with dexamethasone (+DEX) are the averages of two replicates. The presence of RY promoter motifs (CATGCA) in the 500 bp (0.5 K), 1, 000 bp (1 K) and 3, 000 bp (3 K) upstream regions of each gene is included. *p-*values are 3.06 × 10^-4^, 1.77 × 10^-4 ^and 2.57 × 10^-2^, respectively. Click here for file

Additional file 2 **Table S2 Genes downregulated by ectopic FUS3 activation**. Values for fold changes in expression after 2 and 4 days (d) of FUS3 activation with dexamethasone (+DEX) are the averages of two replicates. Genes involved in hormone metabolism or response are in bold. Ethylene response genes that were downregulated less than twofold by FUS3 activation at both 2 and 4 days are in italics. The presence of RY promoter motifs (CATGCA) in 500 bp (0.5 K), 1, 000 bp (1 K) and 3, 000 bp (3 K) upstream regions of each gene is included. *p-*values are 4.27 × 10^-2^, 6.06 × 10^-3 ^and 3.46 × 10^-2^, respectively. Click here for file

Additional file 3 **Figure S1 Phenotypic analysis of wild-type plants exposed to dexamethasone (DEX)**. Wild-type seeds were germinated in 10 μM DEX for 2 days and then transferred to soil. The increase in the blade-to-petiole ratio and the appearance of the abaxial trichomes on leaf 5 is comparable to wild-type profiles shown in Figure 3. **(A) **Ratios of blade-to-petiole lengths of individual wild-type rosette leaves. **(B) **Percentage of wild-type rosettes that formed abaxial trichomes at each leaf position. Click here for file

## References

[B1] Akam M The molecular basis for metameric pattern in the *Drosophila *embryo Development 1987 101 1 22 2896587

[B2] Thummel CS Molecular mechanisms of developmental timing in *C. elegans *and *Drosophila* Dev Cell 2001 1 453 465 10.1016/s1534-5807(01)00060-0 11703937

[B3] Kerstetter RA Poethig RS The specification of leaf identity during shoot development Annu Rev Cell Dev Biol 1998 14 373 398 10.1146/annurev.cellbio.14.1.373 9891788

[B4] Henderson IR Dean C Control of *Arabidopsis *flowering: the chill before the bloom Development 2004 131 3829 3838 10.1242/dev.01294 15289433

[B5] Poethig RS Small RNAs and developmental timing in plants Curr Opin Genet Dev 2009 19 374 378 10.1016/j.gde.2009.06.001 PMC2765200 19703647

[B6] Bäumlein H Miséra S Luerßen H Kölle K Horstmann C Wobus U Müller AJ The *FUS3 *gene of *Arabidopsis thaliana *is a regulator of gene expression during late embryogenesis Plant J 1994 6 379 387

[B7] Keith K Kraml M Dengler NG McCourt P *fusca3*: a heterochronic mutation affecting late embryo development in Arabidopsis Plant Cell 1994 6 589 600 10.1105/tpc.6.5.589 PMC160461 12244252

[B8] Meinke DW Franzmann LH Nickle TC Yeung EC *Leafy cotyledon *mutants of Arabidopsis Plant Cell 1994 6 1049 1064 10.1105/tpc.6.8.1049 PMC160500 12244265

[B9] West MAL Matsudaira Yee K Danao J Zimmerman JL Fischer RL Goldberg RB Harada JJ LEAFY COTYLEDON1 is an essential regulator of late embryogenesis and cotyledon identity in Arabidopsis Plant Cell 1994 6 1731 1745 10.1105/tpc.6.12.1731 PMC160558 12244233

[B10] Nambara E Hayama R Tsuchiya Y Nishimura M Kawaide H Kamiya Y Naito S The role of *ABI3 *and *FUS3 *loci in *Arabidopsis thaliana *on phase transition from late embryo development to germination Dev Biol 2000 220 412 423 10.1006/dbio.2000.9632 10753527

[B11] Gazzarrini S Tsuchiya Y Lumba S Okamoto M McCourt P The transcription factor *FUSCA3 *controls developmental timing in *Arabidopsis *through the hormones gibberellin and abscisic acid Dev Cell 2004 7 373 385 10.1016/j.devcel.2004.06.017 15363412

[B12] Curaba J Moritz T Blervaque R Parcy F Raz V Herzog M Vachon G *AtGA3ox2*, a key gene responsible for bioactive gibberellin biosynthesis, is regulated during embryogenesis by *LEAFY COTYLEDON2 *and *FUSCA3 *in Arabidopsis Plant Physiol 2004 136 3660 3669 10.1104/pp.104.047266 PMC527164 15516508

[B13] Lotan T Ohto M Matsudaira Yee K West MAL Lo R Kwong RW Yamagishi K Fischer RL Goldberg RB Harada JJ *Arabidopsis *LEAFY COTYLEDON1 is sufficient to induce embryo development in vegetative cells Cell 1998 93 1195 205 10.1016/s0092-8674(00)81463-4 9657152

[B14] Stone SL Kwong LW Matsudaira Yee K Pelletier J Lepiniec L Fischer RL Goldberg RB Harada JJ *LEAFY COTYLEDON2 *encodes a B3 domain transcription factor that induces embryo development Proc Natl Acad Sci USA 2001 98 11806 11811 10.1073/pnas.201413498 PMC58812 11573014

[B15] Tsukaya H Shoda K Kim GT Uchimiya H Heteroblasty in *Arabidopsis thaliana *(L.) Heynh Planta 2000 210 536 542 10.1007/s004250050042 10787046

[B16] Kushiro T Okamoto M Nakabayashi K Yamagishi K Kitamura S Asami T Hirai N Koshiba T Kamiya Y Nambara E The *Arabidopsis *cytochrome P450 CYP707A encodes ABA 8'-hydroxylases: key enzymes in ABA catabolism EMBO J 2004 23 1647 1656 10.1038/sj.emboj.7600121 PMC391058 15044947

[B17] Reidt W Wohlfarth T Ellerström M Czihal A Tewes A Ezcurra I Rask L Bäumlein H Gene regulation during late embryogenesis: the RY motif of maturation-specific gene promoters is a direct target of the FUS3 gene product Plant J 2000 21 401 408 10.1046/j.1365-313x.2000.00686.x 10758492

[B18] Mönke G Altschmied L Tewes A Reidt W Mock HP Bäumlein H Conrad U Seed-specific transcription factors ABI3 and FUS3: molecular interaction with DNA Planta 2004 219 158 166 10.1007/s00425-004-1206-9 14767767

[B19] Alonso JM Stepanova AN Leisse TJ Kim CJ Chen H Shinn P Stevenson DK Zimmerman J Barajas P Cheuk R Gadrinab C Heller C Jeske A Koesema E Meyers CC Parker H Prednis L Ansari Y Choy N Deen H Geralt M Hazari N Hom E Karnes M Mulholland C Ndubaku R Schmidt I Guzman P Aguilar-Henonin L Schmid M Weigel D Carter DE Marchand T Risseeuw E Brogden D Zeko A Crosby WL Berry CC Ecker JR Genome-wide insertional mutagenesis of *Arabidopsis thaliana* Science 2003 301 653 657 10.1126/science.1086391 12893945

[B20] Guo H Ecker JR Plant responses to ethylene gas are mediated by SCF^EBF1/EBF2^-dependent proteolysis of EIN3 transcription factor Cell 2003 115 667 677 10.1016/s0092-8674(03)00969-3 14675532

[B21] Potuschak T Lechner E Parmentier Y Yanagisawa S Grava S Koncz C Genschik P EIN3-dependent regulation of plant ethylene hormone signaling by two *Arabidopsis *F box proteins: EBF1 and EBF2 Cell 2003 115 679 689 10.1016/s0092-8674(03)00968-1 14675533

[B22] Schaller GE Kieber JJ Somerville C, Meyerowitz E Ethylene The Arabidopsis Book 2002 1 Rockville, MD: American Society of Plant Biologists e0071 http://www.bioone.org/doi/pdf/10.1199/tab.0071 10.1199/tab.0071 PMC3243340 22303216

[B23] Tsukaya H Somerville C, Meyerowitz E Leaf development The Arabidopsis Book 2002 1 Rockville, MD: American Society of Plant Biologists e0072 http://www.bioone.org/doi/pdf/10.1199/tab.0072 10.1199/tab.0072 PMC3243299 22303217

[B24] Telfer A Bollman KM Poethig RS Phase change and the regulation of trichome distribution in *Arabidopsis thaliana* Development 1997 124 645 654 10.1242/dev.124.3.645 9043079

[B25] Bollman KM Aukerman MJ Park MY Hunter C Berardini TZ Poethig RS HASTY, the *Arabidopsis *ortholog of exportin 5/MSN5, regulates phase change and morphogenesis Development 2003 130 1493 1504 10.1242/dev.00362 12620976

[B26] Lu QS Dela Paz J Pathmanathan A Chiu RS Tsai AYL Gazzarrini S The C-terminal domain of FUSCA3 negatively regulates mRNA and protein levels and mediates sensitivity to the hormones abscisic acid and gibberellic acid in Arabidopsis Plant J 2010 64 100 113 10.1111/j.1365-313X.2010.04307.x 20663088

[B27] Tsuchiya Y Nambara E Naito S McCourt P The *FUS3 *transcription factor functions through the epidermal regulator *TTG1 *during embryogenesis in *Arabidopsis* Plant J 2004 37 73 81 10.1046/j.1365-313x.2003.01939.x 14675433

[B28] Cox MCH Benschop JJ Vreeburg RAM Wagemaker CAM Moritz T Peeters AJM Voesenek LACJ The roles of ethylene, auxin, abscisic acid, and gibberellin in the hyponastic growth of submerged *Rumex palustris *petioles Plant Physiol 2004 136 2948 2960 10.1104/pp.104.049197 PMC523357 15466223

[B29] Kende H van der Knaap E Cho HT Deepwater rice: a model plant to study stem elongation Plant Physiol 1998 118 1105 1110 10.1104/pp.118.4.1105 PMC1539197 9847084

[B30] Kuwabara A Ikegami K Koshiba T Nagata T Effects of ethylene and abscisic acid upon heterophylly in *Ludwigia arcuata *(Onagraceae) Planta 2003 217 880 887 10.1007/s00425-003-1062-z 12844266

[B31] Benschop JJ Jackson MB Gühl K Vreeburg RAM Croker SJ Peeters AJM Voesenek LACJ Contrasting interactions between ethylene and abscisic acid in *Rumex *species differing in submergence tolerance Plant J 2005 44 756 768 10.1111/j.1365-313X.2005.02563.x 16297068

[B32] Hoffmann-Benning S Kende H On the role of abscisic acid and gibberellin in the regulation of growth in rice Plant Physiol 1992 99 1156 1161 10.1104/pp.99.3.1156 PMC1080597 16668983

[B33] Gazzarrini S McCourt P Cross-talk in plant hormone signalling: what Arabidopsis mutants are telling us Ann Bot 2003 91 605 612 10.1093/aob/mcg064 PMC4242347 12714359

[B34] Nishii K Kuwabara A Nagata T Characterization of anisocotylous leaf formation in *Streptocarpus wendlandii *(Gesneriaceae): significance of plant growth regulators Ann Bot 2004 94 457 467 10.1093/aob/mch160 PMC4242185 15286012

[B35] Chiwocha SDS Cutler AJ Abrams SR Ambrose SJ Yang J Ross ARS Kermode AR The *etr1-2 *mutation in *Arabidopsis thaliana *affects the abscisic acid, auxin, cytokinin and gibberellin metabolic pathways during maintenance of seed dormancy, moist-chilling and germination Plant J 2005 42 35 48 10.1111/j.1365-313X.2005.02359.x 15773852

[B36] Ghassemian M Nambara E Cutler S Kawaide H Kamiya Y McCourt P Regulation of abscisic acid signalling by the ethylene response pathway in Arabidopsis Plant Cell 2000 12 1117 1126 10.1105/tpc.12.7.1117 PMC149053 10899978

[B37] LeNoble ME Spollen WG Sharp RE Maintenance of shoot growth by endogenous ABA: genetic assessment of the involvement of ethylene suppression J Exp Bot 2004 55 237 245 10.1093/jxb/erh031 14673028

[B38] Beaudoin N Serizet C Gosti F Giraudat J Interactions between abscisic acid and ethylene signaling cascades Plant Cell 2000 12 1103 1116 10.1105/tpc.12.7.1103 PMC149052 10899977

[B39] Kagaya Y Toyoshima R Okuda R Usui H Yamamoto A Hattori T LEAFY COTYLEDON1 controls seed storage protein genes through its regulation of *FUSCA3 *and *ABSCISIC ACID INSENSITIVE3* Plant Cell Physiol 2005 46 399 406 10.1093/pcp/pci048 15695450

[B40] Lopez-Molina L Mongrand S Chua NH A postgermination developmental arrest checkpoint is mediated by abscisic acid and requires the ABI5 transcription factor in *Arabidopsis* Proc Natl Acad Sci USA 2001 98 4782 4787 10.1073/pnas.081594298 PMC31911 11287670

[B41] Peragine A Yoshikawa M Wu G Albrecht HL Poethig RS *SGS3 *and *SGS2/SDE1/RDR6 *are required for juvenile development and the production of *trans*-acting siRNAs in *Arabidopsis* Genes Dev 2004 18 2368 2379 10.1101/gad.1231804 PMC522987 15466488

[B42] Hunter CA Aukerman MJ Sun H Fokina M Poethig RS *PAUSED *encodes the Arabidopsis exportin-t ortholog Plant Physiol 2003 132 2135 2143 10.1104/pp.103.023309 PMC184644 12913168

[B43] Toufighi K Brady SM Austin R Ly E Provart NJ The Botany Array Resource: e-Northerns, Expression Angling, and promoter analyses Plant J 2005 43 153 163 10.1111/j.1365-313X.2005.02437.x 15960624

[B44] Ogawara T Higashi K Kamada H Ezura H Ethylene advances the transition from vegetative growth to flowering in *Arabidopsis thaliana* J Plant Physiol 2003 160 1335 1340 10.1078/0176-1617-01129 14658386

[B45] Clough SJ Bent AF Floral dip: a simplified method for *Agrobacterium*-mediated transformation in *Arabidopsis thaliana* Plant J 1998 16 735 743 10.1046/j.1365-313x.1998.00343.x 10069079

[B46] Cutler SR Ehrhardt DW Griffitts JS Somerville CR Random GFP::cDNA fusions enable visualization of subcellular structures in cells of *Arabidopsis *at a high frequency Proc Natl Acad Sci USA 2000 97 3718 3723 10.1073/pnas.97.7.3718 PMC16306 10737809

[B47] Donnelly PM Bonetta D Tsukaya H Dengler RE Dengler NG Cell cycling and cell enlargement in developing leaves of *Arabidopsis* Dev Biol 1999 215 407 419 10.1006/dbio.1999.9443 10545247

[B48] Zhu T Budworth P Chen W Provart N Chang HS Guimil S Su G Estes B Zou G Wang X Transcriptional control of nutrient partitioning during rice grain filling Plant Biotechnol J 2003 1 59 70 10.1046/j.1467-7652.2003.00006.x 17147681

[B49] Eisen MB Spellman PT Brown PO Botstein D Cluster analysis and display of genome-wide expression patterns Proc Natl Acad Sci USA 1998 95 14863 14868 10.1073/pnas.95.25.14863 PMC24541 9843981

[B50] DeRisi JL Iyer VR Brown PO Exploring the metabolic and genetic control of gene expression on a genomic scale Science 1997 278 680 686 10.1126/science.278.5338.680 9381177

