# Supplementary material for: The embryonic leaf identity gene FUSCA3 regulates vegetative phase transitions by negatively modulating ethylene-regulated gene expression in Arabidopsis
Source: BMC Biol. 2012 Feb 20;10:8. doi: 10.1186/1741-7007-10-8 (PMC3305478; doi:10.1186/1741-7007-10-8)
Supplement: Additional file 1 — Table S1 Genes upregulated by ectopic FUS3 activation. Values for fold changes in expression after 2 and 4 days (d) of FUS3 activation with dexamethasone (+DEX) are the averages of two replicates. The presence of RY promoter motifs (CATGCA) in the 500 bp (0.5 K), 1, 000 bp (1 K) and 3, 000 bp (3 K) upstream regions of each gene is included. p-values are 3.06 × 10^-4^, 1.77 × 10^-4^ and 2.57 × 10^-2^, respectively. [file 1741-7007-10-8-S1.PDF]

Table S1

AGI	Gene	Annotation (TAIR)	Fold Change		RY		
			2d	4d	0.5K	1K	3K
At5g02760	PP2C	protein phosphatase 2C	2.1	2.2		x	x
At5g57785		unknown protein	2.1	2.8	x	x	x
At4g34250	KCS16	3-ketoacyl-CoA synthase 16	2.2	2.5	x	x	x
At4g19380		long-chain fatty alcohol dehydrogenase	2.2	2.6			
At4g12470		lipid-transfer protein	2.3	1.6	x	x	x
At5g01870		lipid-transfer protein	2.3	1.9		x	x
At5g02230		haloacid dehalogenase-like hydrolase	2.4	2.9			x
At1g06080	ADS1	delta 9 desaturase 1	2.4	1.6	x	x	x
At4g23190	ATRLK3/CRK11	protein kinase	2.6	1.3			x
At5g55620		unknown protein	2.7	2.9	x	x	x
At3g28850		glutaredoxin family protein	2.7	1.1			x
At5g09440	EXL4	EXORDIUM like 4	3.0	2.6	x	x	x
At3g43270		invertase/pectin methylesterase inhibitor	3.1	3.0	x	x	x
At2g38530	LTP2	lipid transfer protein 2	3.1	4.2		x	x
At5g22500	FAR1	fatty acid reductase 1	3.2	1.5			
At1g62290		saposin-like aspartyl protease	3.2	4.6	x	x	x
At5g36910	THI2.2	thionin 2.2	3.3	2.9	x	x	x
At5g66170	STR18	sulfurtransferase 18	3.5	3.3			x
At3g23550		MATE efflux family protein	3.8	8.5	x	x	x
