# Supplementary material for: The embryonic leaf identity gene FUSCA3 regulates vegetative phase transitions by negatively modulating ethylene-regulated gene expression in Arabidopsis
Source: BMC Biol. 2012 Feb 20;10:8. doi: 10.1186/1741-7007-10-8 (PMC3305478; doi:10.1186/1741-7007-10-8)
Supplement: Additional file 2 — Table S2 Genes downregulated by ectopic FUS3 activation. Values for fold changes in expression after 2 and 4 days (d) of FUS3 activation with dexamethasone (+DEX) are the averages of two replicates. Genes involved in hormone metabolism or response are in bold. Ethylene response genes that were downregulated less than twofold by FUS3 activation at both 2 and 4 days are in italics. The presence of RY promoter motifs (CATGCA) in 500 bp (0.5 K), 1, 000 bp (1 K) and 3, 000 bp (3 K) upstream regions of each gene is included. p-values are 4.27 × 10^-2^, 6.06 × 10^-3^ and 3.46 × 10^-2^, respectively. [file 1741-7007-10-8-S2.PDF]

Table S2

AGI	Gene	Annotation (TAIR)	Fold Change		RY		
			2d	4d	0.5K	1K	3K
At3g56980	ORG3	basic helix-loop-helix DNA-binding protein	0.3	0.6			x
At2g39650	DUF506	unknown function	0.3	0.4			
**At4g11280**	**ACS6**	**ACC synthase 6**	**0.3**	**0.3**			
At1g47400		unknown protein	0.4	0.8			
At1g04770		tetratricopeptide repeat protein	0.4	0.4		x	x
At3g57490		ribosomal protein S5 family protein	0.4	0.9	x	x	x
**At5g61600**	**ERF104**	**ethylene response factor 104**	**0.4**	**0.5**		**x**	**x**
At3g56400	WRKY70	WRKY DNA-binding protein 70	0.4	0.3			x
At3g09440	Hsp70	heat shock protein 70	0.4	0.6		x	x
At3g20590		LEA hydroxyproline-rich glycoprotein	0.4	1.1	x	x	x
At2g33850		unknown protein	0.4	0.3		x	x
At1g60590		pectin lyase-like superfamily protein	0.4	0.5			x
At4g04610	APR1	APS reductase 1	0.4	0.6		x	x
**At4g38860**		**SAUR-like auxin-responsive protein**	**0.4**	**0.9**	**x**	**x**	**x**
At2g16060	AHB1	non-symbiotic hemoglobin 1	0.4	0.8			x
At1g73500	ATMKK9	MAP kinase kinase 9	0.5	0.6		x	x
**At1g13260**	**EDF4**	**ethylene response DNA binding factor 4**	**0.5**	**0.7**	**x**	**x**	**x**
At5g49480	ATCP1	calcium binding protein 1	0.5	0.7		x	x
At2g27420		cysteine proteinase	0.5	0.6			
At4g27280		calcium-binding EF-hand family protein	0.5	0.4		x	x
At1g17170	ATGSTU24	glutathione S-transferase	0.5	0.7			x
At2g39705	DVL11	Rotundifolia-like 8	0.5	0.6			
At1g51080		unknown protein	0.5	0.7			x
**At5g45340**	**CYP707A3**	**cytochrome P450**	**0.5**	**0.3**	**x**	**x**	**x**
At5g37990		SAM-dependent methyltransferase	0.5	1.0			x
**At5g25190**	**ESE3**	**ethylene and salt inducible 3**	**0.5**	**0.4**			**x**
At4g24570	DIC2	dicarboxylate carrier 2	0.5	0.4			x
**At3g50060**		**R2R3 transciption factor**	**0.5**	**0.4**	**x**	**x**	**x**
At3g05890	RCI2B	Low temperature and salt responsive prote	0.5	0.5			
**At5g37770**	**TCH2/CML24**	**EF hand calcium-binding protein**	**0.5**	**0.6**			
At4g29030		putative membrane lipoprotein	0.5	0.2			x
At5g26260		TRAF-like family protein	0.5	0.9	x	x	x
**At4g17500**	**ERF1**	**ethylene responsive factor 1**	**0.5**	**0.7**			**x**
At3g06070		unknown protein	0.5	0.7	x	x	x
At5g47220	***ERF2***	***ethylene responsive factor 2***	*0.8*	*0.7*		*x*	*x*
At1g68840	***EDF2***	***ethylene response DNA binding factor 2***	*0.6*	*0.7*	*x*	*x*	*x*
At1g25560	***EDF1***	***ethylene response DNA binding factor 1***	*0.6*	*0.6*	*x*	*x*	*x*
