# Supplementary figures and images for: The embryonic leaf identity gene FUSCA3 regulates vegetative phase transitions by negatively modulating ethylene-regulated gene expression in Arabidopsis
Source: BMC Biol. 2012 Feb 20;10:8. doi: 10.1186/1741-7007-10-8 (PMC3305478; doi:10.1186/1741-7007-10-8)

Figure S1

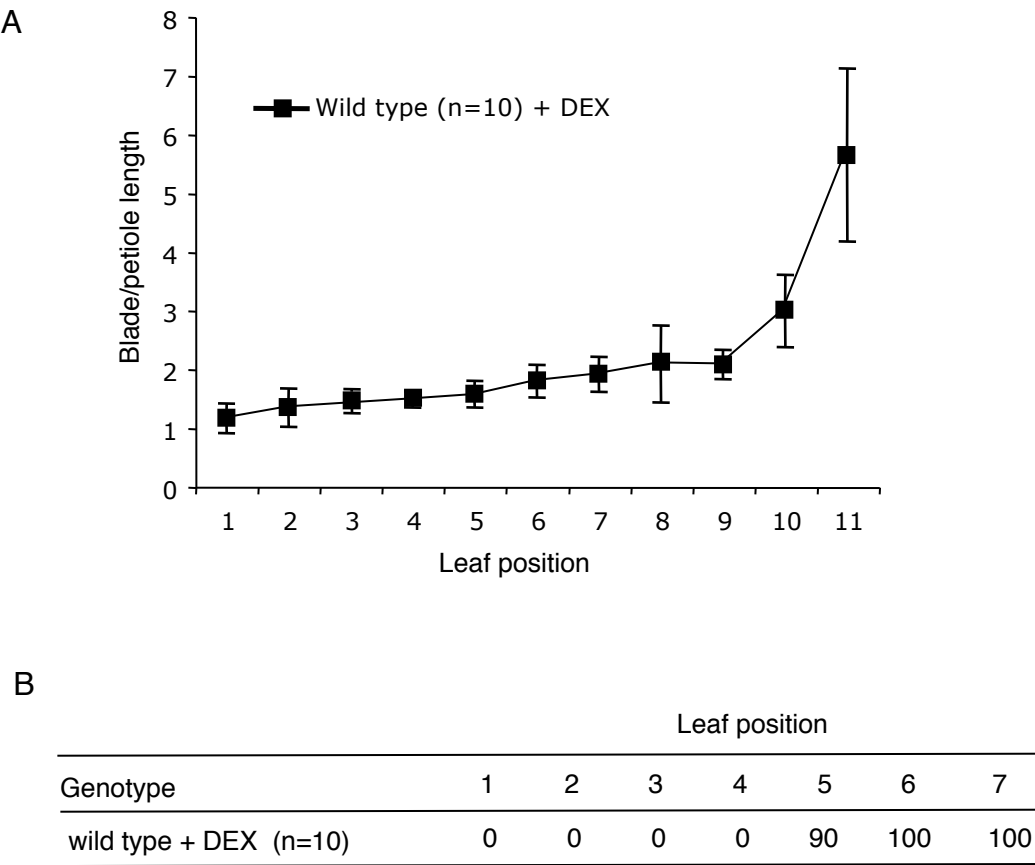

Supplement: Additional file 3 — Figure S1 Phenotypic analysis of wild-type plants exposed to dexamethasone (DEX). Wild-type seeds were germinated in 10 μM DEX for 2 days and then transferred to soil. The increase in the blade-to-petiole ratio and the appearance of the abaxial trichomes on leaf 5 is comparable to wild-type profiles shown in Figure 3. (A) Ratios of blade-to-petiole lengths of individual wild-type rosette leaves. (B) Percentage of wild-type rosettes that formed abaxial trichomes at each leaf position. [file 1741-7007-10-8-S3.PDF]
